# Mesenchymal stem cell therapy for pulmonary hypertension: an update

**DOI:** 10.3389/fbioe.2025.1692396

**Published:** 2025-11-25

**Authors:** Renmei Tian, Shangfu Xu, Chan Liu, Yuying Wang, Yutong Wu, Lisheng Li, Juan Liu

**Affiliations:** 1 Key Laboratory of Cell Engineering of Guizhou Province, Affiliated Hospital of Zunyi Medical University, Zunyi, Guizhou, China; 2 Key Laboratory of Basic Pharmacology of Ministry of Education and Joint International Research Laboratory of Ethnomedicine of Ministry of Education, Zunyi Medical University, Zunyi, Guizhou, China; 3 Department of Pharmacology, Key Laboratory of Basic Pharmacology of Guizhou Province and School of Pharmacy, Zunyi Medical University, Zunyi, Guizhou, China; 4 Guizhou Biomanufacturing Laboratory, Affiliated Hospital of Zunyi Medical University, Zunyi, Guizhou, China

**Keywords:** mesenchymal stem cell, pulmonary hypertension, exosome, endothelial mesenchymal transition, signaling pathways, microRNA

## Abstract

Pulmonary Hypertension (PH) is a life-threatening condition characterized by pulmonary vascular remodeling. Without treatment, it may progress to right heart failure, the main cause of death in such cases. Although traditional drugs can slow PH progression, their efficacy is often limited, underscoring an urgent need for new treatments. Stem cell therapy, meanwhile, has emerged as a promising approach for various refractory diseases, with considerable therapeutic potential. This article reviews preclinical findings on mesenchymal stem cell (MSC) therapy for PH, focusing on how MSCs alleviate right ventricular failure, reverse pulmonary arterial smooth muscle cell proliferation, restore endothelial function, and regulate anti-inflammatory factor expression. It also discusses the therapeutic effects of MSC-derived exosomes. The current challenges and future perspectives of MSCs in clinical applications are also explored. Preclinical studies suggest that MSCs hold considerable promise for treating PH. However, further studies are needed to clarify the mechanisms behind their therapeutic effects and develop strategies for the safe, efficient large-scale production of MSCs for clinical use.

## 1 Introduction

**FIGURE 1:**
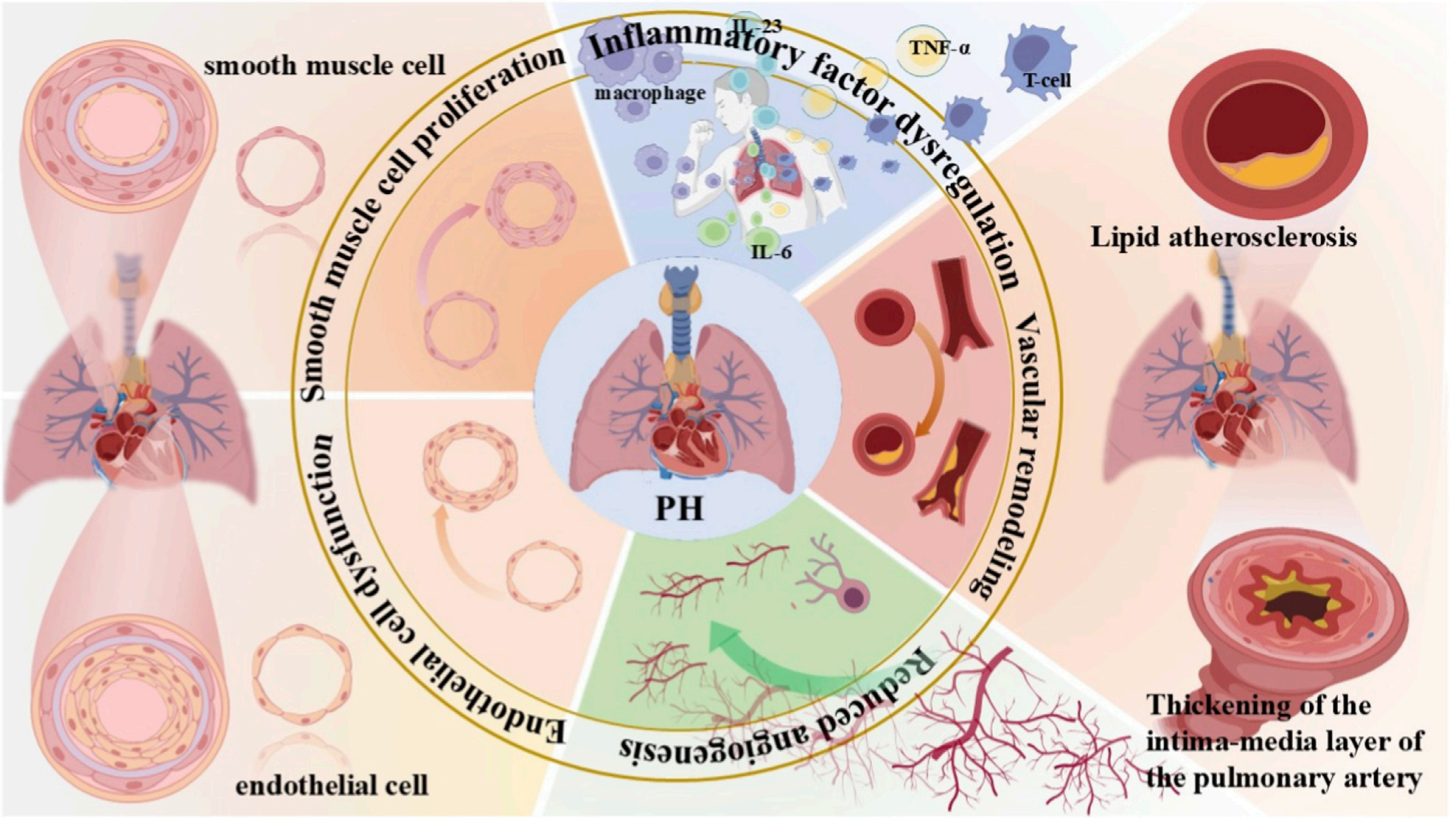
Overview of the pathogenesis of pulmonary hypertension. Due to disease triggers, dysregulation of inflammatory factors such as IL-23, IL-6, and TNF-α released by macrophages and T cells induces chronic inflammation in lung tissues. Lipid atherosclerosis and thickening of the intima-media layer of pulmonary arteries lead to abnormal pulmonary vascular structure and lumen stenosis. Dysregulated pulmonary angiogenesis impairs pulmonary circulation, thereby accelerating the progression of PH. Dysfunction and increased apoptosis of endothelial cells disrupt the normal function of pulmonary vascular endothelium, ultimately resulting in ECs with anti-apoptotic and pro-angiogenic properties. Excessive proliferation of smooth muscle cells causes vascular wall thickening and lumen narrowing. These mechanisms interact synergistically to drive the pathological progression of PH. PH, pulmonary hypertension.

Pulmonary hypertension (PH) is a pathophysiological condition characterized by abnormally elevated pulmonary artery pressure, resulting from various known or unknown causes. The clinical hemodynamic diagnostic standards for PH specify a mean pulmonary artery pressure of ≥20 mmHg, as measured via right heart catheterization under resting conditions at sea level (Simonneau et al., 2019). PH is mainly distinguished by pulmonary vascular restructuring, along with right ventricular hypertrophy and failure. Pulmonary vascular remodeling encompasses several key processes: dysfunction of pulmonary artery endothelial cells, hyperproliferation of pulmonary artery smooth muscle cells, muscularization of distal small pulmonary arteries, deposition of extracellular matrix proteins and perivascular inflammation (Crosswhite and Sun, 2014; Jia et al., 2023) (Figure 1). If not treated promptly, right ventricular failure may occur, the top cause of death in PH patients. Further progress in pulmonary hypertension treatment over the past decade notwithstanding, its long-term prognosis remains unsatisfactory, with a mere 61% 5-year survival rate among patients on medication (Boucly et al., 2021), and the mortality rate is relatively high. The primary treatment approaches currently used for PH include nitric oxide, prostacyclin, calcium channel blockers and endothelin receptor antagonists (Kondo et al., 2019). The approved drugs for pulmonary arterial hypertension (PAH) treatment primarily include nifedipine, sildenafil, bosentan, beraprost, and imatinib, among others (Auth and Klinger, 2023). Possible therapeutic targets for PAH encompass vasoactive peptides, oxidative stress, tyrosine kinases, Rho kinases, and metabolic pathways, in addition to anti-inflammatory medications and gene therapy—all of which are presently being actively explored (Zolty, 2020). Although these medications can improve patients’ exercise capacity, quality of life, and pulmonary circulation hemodynamics, they do not provide a cure for PH. Consequently, there is an urgent need for highly effective emerging therapeutic modalities.

For more than a decade, cell therapy has emerged as a treatment option for a variety of incurable diseases. Stem cells, as an important representative of cell therapy, have become a research hotspot of increasing interest to researchers in recent years. Several cell types, such as MSCs, induced pluripotent stem cells and endothelial progenitor cells, show promising therapeutic effects (Sun and Sun, 2022). In particular, MSCs are distinguished by their multilineage differentiation potential, pro-angiogenic properties, immunomodulatory effects, robust expansion capacity *in vitro*, and ease of procurement from adult tissues (Granton et al., 2015). Most importantly, their low immunogenicity has led to widespread acceptance in clinical practice (Wang et al., 2007; Lewis et al., 2024). MSCs offer a new method for treating PH. Findings reinforce the potential therapeutic benefits of MSCs, which alleviate PH development by ameliorating endothelial and smooth muscle cell hyperproliferation and reducing inflammatory factor secretion (Zhong et al., 2024), and the hemodynamic and histologic progression of PAH was significantly attenuated (Zhang et al., 2012). This review outlines preclinical findings on MSC therapy for PH, focusing on how MSCs reverse pulmonary artery smooth muscle cell proliferation, repair endothelial dysfunction, reduce inflammatory factor expression, and ease right ventricular failure. Additionally, it also explores the therapeutic efficacy of exosomes, along with the challenges and future prospects of MSC application in PH.

## 2 The source, characteristics and roles of MSCs

Fridenshtein et al. published the first study on MSCs in 1966, in which they cultured osteoblasts from guinea pig bone marrow and spleen cells (Friedenstein et al., 1966). Caplan first coined the term “mesenchymal stem cells” in 1991, utilizing the self-repairing ability of cells as a new therapeutic technique. Initially, MSCs were derived from bone marrow, hence the name bone marrow stromal cells. Subsequently, cytophenotypic characterization revealed these cells to be spindle-shaped stromal cells derived from multiple tissue types, distinguished by their capacity for self-renewal and multilineage differentiation (Pittenger et al., 1999).

**FIGURE 2:**
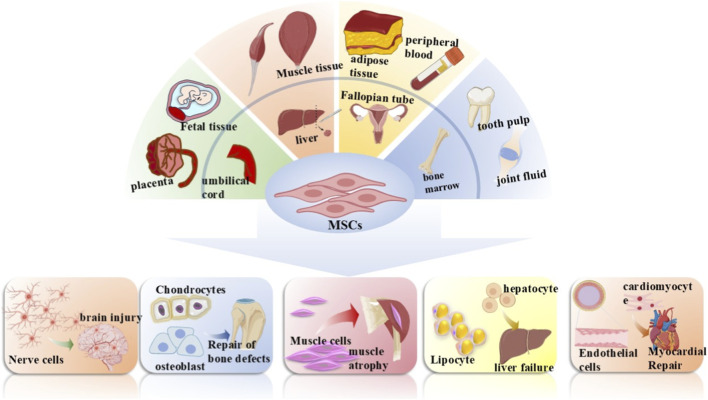
Mesenchymal stem cells (MSCs) source and differentiation. MSCs can be isolated from a variety of human tissues, including the placenta, fetal tissue, umbilical cord, muscle tissue, liver, adipose tissue, fallopian tube, peripheral blood, bone marrow, dental pulp, and synovial fluid. Under specific conditions, they are capable of differentiating into nerve cells, chondrocytes, osteoblasts, muscle cells, hepatocytes, adipocytes, cardiomyocytes, and endothelial cells, which can be applied to the repair and treatment of multiple diseases such as brain injury repair, bone defect repair, and myocardial repair.

Currently, MSCs can be extracted from a variety of tissues, including muscle, bone marrow, placenta, fat, amniotic fluid, umbilical cord, peripheral blood, and fetal tissues. The first transplantation of bone marrow cells revealed their differentiation into osteoblasts, thereby unlocking the potential for multi-lineage differentiation of MSCs (Friedenstein et al., 1966) (Figure 2). Since then, various human-derived MSCs have been used to generate terminally differentiated MSC populations, such as differentiating into adipocytes, osteoblasts, chondrocytes, and other versatile cells (Dominici et al., 2006). Cells from different sources exhibit variations in their self-renewal potential, differentiation capacity (Zheng et al., 2017) and potency of isolated MSCs (Brown et al., 2019). Additionally, the proliferation and differentiation capacities of MSCs from the same tissue can vary with the extraction site (Cao et al., 2024; Deng et al., 2024a). In recent decades, mounting research has demonstrated that MSCs show promising therapeutic effects in treating various diseases in animal models. Their diverse sources and functions have facilitated their widespread application in clinical treatments. Clinical trials investigating MSCs’ therapeutic potential for various diseases are underway, and their safety has been verified (Lee et al., 2019; Cheng et al., 2022; Bydon et al., 2024).

To promote a clearer comprehension and uniform application of MSCs, the International Society for Cellular Therapy (ISCT) set forth standards for their isolation and characterization in 2006 (Dominici et al., 2006). These standards comprise three key aspects: (1) plastic adherence; (2) the ability to differentiate into chondrocytes, osteoblasts and adipocytes under standard culture conditions; and (3) the presence of specific cell surface markers like CD73, CD105, and CD90, alongside the absence of CD79a, CD34, CD11b, CD14, CD45, CD19, and HLA-DR surface molecules.

## 3 Application and mechanisms of MSCs in PH

### 3.1 Application of MSCs in PH

In particular, the application of MSCs in treating PH has emerged as a prominent focus in regenerative medicine research in recent years. The mechanisms underlying their therapeutic effects primarily encompass anti-inflammatory actions, promotion of vascular regeneration, inhibition of pulmonary vascular remodeling, and enhancement of right ventricular function (Sun and Sun, 2022; Zheng et al., 2023a). Earlier studies have documented cases of stem cell therapy for PH. For instance, a 2006 study conducted by Deng et al. reported that bone marrow-derived MSCs could inhibit the proliferation of smooth muscle cells—this finding thereby underscores the therapeutic potential of such cells for cardiovascular diseases, including PH (Deng et al., 2006). Over the past decades, there have been numerous reports on using MSCs to treat PH, with ample preclinical evidence confirming their therapeutic efficacy for the condition (Table 1). All experimental findings consistently demonstrate that the therapeutic efficacy of MSCs for PH is regulated by three critical factors. The first is the influence of MSC sources. A study compared the therapeutic effects of MSCs derived from multiple origins in MCT-induced PH rats, revealing that MSCs from different sources—including ADSCs, BMSCs, and HUC-MSCs—all exert therapeutic effects on PH. Notably, the results indicated that HUC-MSCs exhibit more prominent therapeutic efficacy compared to the other two MSC types (Oh et al., 2021). This is mainly reflected in the more prominent therapeutic advantages of HUC-MSCs in PAH, including improving right ventricular function, inhibiting pulmonary vascular remodeling, enhancing pulmonary colonization, regulating immuno-inflammatory responses, and repairing relevant signaling pathways (Lee et al., 2015; Liu et al., 2024b). In addition to the varying therapeutic effects of stem cells from different sources on PH, different administration routes are also closely associated with the therapeutic outcomes of PH. Currently, preclinical studies mainly focus on three administration routes: peripheral intravenous injection, intratracheal (IT) injection, and intracardiac (IC) injection. These three distinct routes significantly influence the therapeutic efficacy of stem cells by regulating their homing ability, pulmonary retention, and systemic biodistribution after transplantation *in vivo* (Table 2). The third factor encompasses the selection of administration dosage, timing, and frequency, for which a substantial body of preclinical studies has currently provided evidence supporting rational optimization. Regarding dosage, it needs to be precisely determined based on the specific PH model and intervention substance—low doses (e.g., MSC-EVs at 20 μg/kg protein content or MSCs at 3 × 10^5^ cells per animal) can often achieve significant therapeutic effects comparable to high doses while offering greater advantages for clinical translation (Lee et al., 2017; Klinger et al., 2021). In terms of frequency, short-term high-frequency administration (e.g., once daily for 3 consecutive days) enables rapid initiation of therapeutic efficacy, whereas long-term administration once weekly or once every 2 weeks is sufficient to maintain stable improvements, which aligns with clinical practice requirements (Klinger et al., 2021). For administration timing, intervention initiated in the early stage of the disease or at the peak of vascular injury can maximally block pathological progression and yield the optimal therapeutic outcome; in contrast, delayed administration may require an increased dosage to achieve equivalent efficacy due to the higher resistant (Chou et al., 2016; Lee et al., 2017; Klinger et al., 2021). Drawing on the research data and theoretical foundations accumulated through a series of preliminary experiments, a highly groundbreaking clinical study was formally published in 2022. Hansmann and colleagues achieved successful treatment of a 3-year-old child with severe PAH using conditioned medium from human umbilical cord MSCs. After five treatment sessions (Two intratracheal injections and three peripheral intravenous injections), and no repeat infusion in 2–4 months, the patient showed significant enhancements in exercise tolerance, cardiovascular function, and inflammatory markers, with no adverse reactions reported (Hansmann et al., 2022). This represents the first reported successful case of using HUCMSC-CM in the treatment of PAH, thereby laying the groundwork for future clinical trials. Drawing on a wealth of experimental data from the past 10 years, MSCs have proven to be safe, positioning them as a promising therapeutic option for PH.

**TABLE 1:** Literature on MSC therapy for PH in the last decade.

Cell source	Application form	Animal model	Route and dosage	Treatment course	Major findings	Reference
BMSCs	EVs	Rats and hyperoxia for 2 weeks	It and 12 × 10^8^ BM-MSC and WJ-MSC EVs suspended in 50 µL saline	Treat on day 3, execute on day 14 or week 3	EV from MSC has potential therapy for BPD-PH, and WJ-MSC and BM-MSC have similar therapeutic effects on BPD-PH	Sharma et al. (2022)
BMSCs	EVs	Mice and hyperoxia for 18 days	Iv and 1.5 ×10^10^ MSC-EVs every 3 days	Treatment in day 18, execution in day 27	GA-EVs significantly enhance the therapeutic efficacy, both *in vitro* and *in vivo*, through improved targeted delivery to diseased PASMCs for improving vascular remodeling	Zhu et al. (2024a)
BMSCs	Exo	Rats and 3 weeks SuHx (25 mg/kg)	Iv and 25 µg MSC- Exo three times a week	Treatment in week 3, execution in week 4	MSC-Exo can mitigate hypoxia-induced proliferation and migration of smooth muscle cells by influencing the secretion of endothelial exosomes	Liu et al. (2024d)
HUC-MSCs	Exo	Rats and 3 weeks MCT (50 mg/kg)	Iv and 50 µg MSC- Exo in 200 μL pbs	Treatment in week 3, execution in week 4	UC-MSC-Exo could significantly attenuate MCT-induced PH and right ventricular hypertrophy	Zhang et al. (2024c)
HUC-MSCs	NVs	Rats and 4 weeks MCT (60 mg/kg)	Iv and 500 μL/kg body weight (NVs concentration of 1 μg/μL)	Treatment in week 2, execution in week 4	Compared with MSC-EV, MSC-NV significantly inhibited the proliferation, migration and phenotypic switching of PASMCs, which resulted in more efficacy and advantages for PH	Hu et al. (2022)
HUC-MSCs	Exo	Rats and 3 weeks MCT (50 mg/kg)	Intravenous and 25 μg/d MSC-Exo	Treatment in week 3, execution in week 4	MSC-Exo administration significantly reduces right ventricular systolic blood pressure and RVHI and inhibits pulmonary vascular remodeling and endothelial-mesenchymal transition processes	Ge et al. (2021)
HUC-MSCs	Exo	Rats and 3 weeks MCT (60 mg/kg)	Iv and 25 μg/day MSC-Exo	Treatment in week 3, execution in week 4	MSC-Exo injection attenuates pulmonary vascular remodeling to alleviate PH progression by regulating RhoA and GSK3β/β-catenin signaling pathways	Zhang et al. (2020a)
HUC-MSCs	Cells	Mice and hyperoxia for 3 weeks	Iv and 1 × 10^6^ MSC	Treat on day 1, execute on day 21	MSC treatment suppresses hypoxia-induced PH in mice, and alterated gut microbiota may play a role in the development and progression of PH	Luo et al. (2021)
ADSCs	Cells	Rats and 2-week MCT (60 mg/kg)	Right jugular vein injection and 3 × 10^6^ ASCs or bFGF-ASCs (cells diluted in 200 μL PBS)	Treatment in week 2, execution in week 4	bFGF-transfected ASCs improved the proliferation of HPAECs and increased the survival of HPAECs by activating the PI3k/Akt pathway	Wang et al. (2019a)
BMSCs	EVs	Rats and 3 weeks SuHx (25 mg/kg)	Iv and 100 μg/kg MSC-EV in 500 µL PBS	Treatment in week 3, execution in week 4 and 5	MSC-EV are effective at preventing and reversing increases in pulmonary artery pressure, right ventricular hypertrophy, and pulmonary vascular remodeling in rats with Su/Hx-induced PH.	Klinger et al. (2020)
BMSCs	Cells	Rats and 3 weeks SuHx (25 mg/kg)	Epicardial placement of a bioscaffold and 2 × 10^6^cells were suspended in 50 µL of Plasmalyte A	Treatment in week 1, execution in week 4	The degrees of cardiomyocyte hypertrophy and RV fibrosis were reduced, and coronary perfusion was increased by the MSC-seeded bioscaffold treatment	Schmuck et al. (2019)
BMSCs	MVs	Rats and 5 weeks MCT (50 mg/kg)	Iv and 30 μg MVs in 0.5 mL saline	Treatment in week 3, execution in week 5	BMMSCs-MVs can alter the balance of the renin angiotensin system (RAS) and reverse pulmonary vascular and right ventricular remodeling thereby alleviating PAH.	Liu et al. (2018)
HUC-MSCs	Cells	Mice and 4 weeks SuHx (20 mg/kg)	Iv and 5 × 10^5^ in 50 μL of vehicle (0.6 U/mL DNaseI in PBS)	Treatment in week 2, execution in week 4	UC-MSCs administration alleviated tissue inflammation and damage, abnormal cell proliferation, and normalized the expression of abnormal factors in PAH	Alencar et al. (2018)
BMSCs	Cells	Rats and 2 weeks MCT (60 mg/kg)	Iv and 2.5 x 10^5^ in 200 μL PBS	Treatment in week 2, execution in week 4	Enhanced migration, self-renewal capacity and anti-inflammatory activity of C1P-treated MSCs improve the therapeutic efficacy of MSCs against PAH	Lim et al. (2016)
HUC-MSCs	CM	Rats and 3 weeks MCT (60 mg/kg)	Iv and 500 μL MSC-CM	Treatment on days 5, 6, 7, 8 and 9, execution on week 3	MSC-CM ameliorates hemodynamic and lung histologic abnormalities in MCT-induced PH and exerts immunosuppressive effects on PH	Liu et al. (2016)
HUC-MSCs	Cells	Rats and 5 days MCT (60 mg/kg)	Iv and 1.0 × 10^6^ cells	Treatment on day 5, execution on day 21	MSCs administration inhibits TNF-α production and downregulates CaN and NFATc2 expression in pulmonary arteries and successfully prevents PASMC hyperproliferation to attenuate PH.	Liu et al. (2015)
HUC-MSCs	Cells	Rats and 1-week MCT (60 mg/kg)	Extra jugular vein injection and 3 × 10^6^ cells	Treatment on day 7, execution on week 3, 4 and 5	hUCB-MSCs administration restored PAH-induced aberrant immunomodulatory behavior and reduced the expression of inflammatory factors thereby ameliorating PAH	Lee et al. (2015)

ADSCs, Adipose mesenchymal stem cells; BMSCs, bone marrow mesenchymal stem cells; BPD, bronchopulmonary dysplasia; bFGF, Basic fibroblast growth factor; CM, conditioned medium; EVs, ectodermal vesicle; EXO, exosome; GA, glucuronic acid; HUC-MSCs, Human Umbilical cord mesenchymal stem cells; HPAECs, human pulmonary artery endothelial cells; MV, microvesicle; MCT, monocrotaline; NVs, nano-vesicle; PASMC, Pulmonary artery smooth muscle cells; RVHI, right ventricular hypertrophy index; SuHx, SU5416 combined with hypoxia.

**TABLE 2:** Different modes of administration affect the distribution of stem cells into the body.

Injection method	Cellular homing	Intrapulmonary retention time	Distribution of systematic organisms	Reference
Peripheral intravenous (IV)	30%–50%	7–14 days	In addition to the lungs, about 20%–30% of MSCs are found in the liver, spleen, bone marrow and, to a lesser extent, in the kidneys	Gholamrezanezhad et al. (2011), Wang et al. (2011), Huang et al. (2022)
Intratracheal (IT)	60%–80%	14–21 days	Systemic distribution is minimal (<5%), only trace amounts of MSCs are absorbed into the bloodstream through the lungs and are mainly confined to the lungs, with little effect on other organs of the body, making it suitable for scenarios in which systemic side effects need to be avoided	Badri et al. (2011), Tolomeo et al. (2023)
Intracardiac (IC)	35%–55%	7–10 days	Approximately 10%–20% of MSCs distribute in the liver and spleen, with no significant risk of systemic dissemination	Wang et al. (2010), Xu et al. (2023)

IV, intravenous; IT, intratracheal; IC, intracardiac; MSCs, mesenchymal stem cells.

### 3.2 Inhibition of smooth muscle cell proliferation and pulmonary vascular remodeling

Pulmonary artery smooth muscle cells (PASMCs), which are mainly situated in the media of pulmonary arteries, control the contraction and relaxation of these vessels and sustain normal pulmonary circulation. Uncontrolled proliferation of PASMCs is a key factor in PH-associated lumen stenosis and vascular remodeling. As a result, targeting such unregulated proliferation represents an effective approach to mitigating PH progression. Recent research has indicated that nuclear factor kappa-B (NF-κB) signaling is critical to PASMCs proliferation and vascular remodeling. Inhibition of NF-κB signaling can reduce PASMCs proliferation, thereby attenuating vascular remodeling (Ogbozor et al., 2015; Patel et al., 2017). In the monocrotaline pyrrole (MCTP)-induced PAH cell model, NF-κB-related protein expression—evidenced by the p-p65/p65 and p-IκBα/IκBα ratios—was significantly heightened. However, treatment with TIPE2-transfected ADSCs reversed these effects. These results indicate that the working mechanism entails suppressing MCTP-induced proliferation and defective apoptosis of PASMCs via the NF-κB signaling pathway, while facilitating the shift of PASMCs from a synthetic to a contractile phenotype (Li et al., 2024a). MSCs secrete high levels of TNF-α to activate the expression of P53 and NF-κB, which inhibits the proliferative capacity of PASMCs by inducing cell cycle arrest through the downregulation of P21, CDK2, and CDK4, thereby mitigating the progression of PH (Liu et al., 2020b). Mounting evidence has confirmed that excessive activation of endothelial nitric oxide synthase is closely tied to PH development, and preserving its homeostasis has been shown to reduce mean pulmonary arterial pressure in rats with PAH (Li et al., 2025). Thus, overexpressing Klotho—a senescence-inhibiting protein—in transplanted MSCs mitigates monocrotaline (MCT)-induced pulmonary vascular remodeling and PASMCs proliferation; this effect is likely mediated by the restoration of endothelial nitric oxide synthase (eNOS) activity (Varshney et al., 2016). Likewise, HUC-MSC-derived exosomes mediating apelin gene therapy exerts therapeutic effects through the PI3K/AKT/eNOS and ERK1/2 signaling pathways. This method effectively lowers pulmonary arterial pressure, eases pulmonary vascular remodeling, and regulates apoptosis in MCT-induced PH rats (Wang et al., 2025).

In addition, the inhibition of PASMC proliferation by MSCs involves multiple signaling pathways. Cheng et al. demonstrated that BMSCs modified with insulin-like growth factor binding protein 3 (IGFBP-3) can suppress the proliferative activity of human PASMCs and induce their apoptosis by activating the PI3K/Akt and Ras-mitogen-activated protein kinase (MAPK) signaling pathways (Cheng et al., 2017a). Notably, MSCs engineered to overexpress let-7a have been shown to alleviate PAH progression by inhibiting PASMC growth through the STAT3-BMPR2 signaling cascade, thereby supporting a promising therapeutic strategy for PAH patients (Cheng et al., 2017b).

### 3.3 Repair of endothelial dysfunction

The vascular endothelium plays a key role in preserving multi-organ health and *in vivo* homeostasis, including the dynamic balance of vasodilation and vasoconstriction (Melikian et al., 2009), as well as angiogenesis and anti-angiogenesis (Vanhoutte, 1989). Pulmonary artery endothelial cell proliferation and dysfunction are significant drivers of PH progression; accordingly, reestablishing normal endothelial function represents a universal aim of current therapeutic approaches (Thenappan et al., 2018). MicroRNAs are a category of non-coding RNAs featuring a 22-base sequence, which exert regulatory effects on gene expression via post-transcriptional modification of target mRNAs (Makeyev and Maniatis, 2008), studies have indicated that microRNAs participate in numerous key biological processes, including cell proliferation and apoptosis (Huber et al., 2015; Yuan et al., 2019). In recent years, It has been found that epigenetic dysregulation of microRNAs plays a role in the pathogenesis of PAH (Feng et al., 2024). Consequently, intervening in the cellular activities of miRNAs has become an important strategy for alleviating PH (Niu et al., 2022). Transplantation of ADSCs attenuates PAH progression in a MCT-induced rat model, which may involve six dysregulated microRNAs (miR-133a-3p,miR-206, miR-200a-3p, miR-141-3p, miR-1246 and miR-537) in ADSCs, They improve pulmonary vascular endothelial remodeling by regulating multiple signaling pathways, including Wnt, VEGF, cytokine-cytokine receptor interactions, mitogen-activated protein kinase, actin cytoskeleton regulation, TGF-β, and p53 signaling cascades (Wang et al., 2019b). A recent study confirmed that miR-191 is one of the circulating miRNAs upregulated in subjects with PH, and its overexpression is associated with the formation of PH (He and Zhang, 2019). Transplanting ADSCs into PAH rats blocks the degradation of BMPR2 and inhibits the expression of miR-191, thereby ameliorating the proliferation of pulmonary artery endothelial cells (Zhang et al., 2019).

Endothelial-mesenchymal transition (EMT) refers to a process where endothelial cells lose their distinctive traits, take on a mesenchymal-like phenotype, and convert into mesenchymal cells (Coll-Bonfill et al., 2015). A growing body of research has indicated that the EMT process is activated in PH rat models induced by MCT or hypoxia (Ranchoux et al., 2015), this indicates that PAH formation is closely intertwined with EMT and that EMT could play a role in PAH progression (Good et al., 2015). Therefore, intervening in the EMT process is an important strategy to alleviate PAH. In both chronic hypoxia-induced and Su5416 hypoxia-induced PH rat models, injecting bone marrow-derived MSCs led to a rise in pulmonary vascular stromal cell markers (vimentin and fibronectin 1) and a drop in endothelial cell markers (platelet endothelial cell adhesion molecule-1 and vascular endothelial cadherin), these changes led to improvements in pulmonary vascular collagen deposition, luminal thickening, and fibrosis (Huang et al., 2020). Compared with normal BMSCs, BMSCs pretreated with different doses of erythropoietin (EPO) more significantly inhibited pulmonary vascular EMT in PH rats. This was mainly evident in the marked decrease in the expression levels of endothelial cell markers (CD31 and VE-cad) and the marked increase in those of mesenchymal cell markers (fibronectin 1 and vimentin) (Zhong et al., 2024). In addition, MSCs may exert an inhibitory effect on EMT by regulating core transcription factors such as Snail and Twist1. Research findings have demonstrated that MSC-derived exosomes can significantly downregulate the expression of ZEB1, Snail1, and the mesenchymal marker vimentin in TGF-β-treated gastric cancer AGS cells, thereby reversing TGF-β-induced EMT (Mirzaei et al., 2023). In a PH model, a study published in ATS Journals revealed that the protein expression levels of EMT-related transcription factors, such as Snail and Twist1, were significantly upregulated in lung tissues of the PH model group. In contrast, following MSC treatment, the abnormal expression of these EMT-related molecules was significantly reversed, accompanied by marked improvements in pulmonary vascular remodeling and pulmonary arterial pressure (Huang et al., 2020). These findings suggest that MSCs attenuate EMT in the pulmonary vascular endothelial cells of the PH model rats.

### 3.4 Attenuates inflammatory factors

Inflammation is a prevalent cellular process that can be categorized as chronic or acute, as well as systemic or localized (Arulselvan et al., 2016; Dolmatova et al., 2021). The development of PH is associated with chronic, localized inflammation, which plays a key role in the progression of pulmonary vascular remodeling (Wang et al., 2021). The renin-angiotensin system has been shown to be involved in the development of PH (Shenoy et al., 2011). Additionally, angiotensin-converting enzyme 2 (ACE2) is a cardioprotective enzyme within the renin-angiotensin system, and its overexpression mitigates chronic hypoxia-induced PH (Oliveira et al., 2023). Thus, ACE2 plays a critical role in cardiopulmonary homeostasis through its vasoprotective function, and the ACE2/Ang-(1–7)/Mas 1 receptor signaling pathway is anticipated to be a promising therapeutic target for PAH (Papavassiliou et al., 2023). Following transplantation of human amniotic mesenchymal stem cells (hAMSCs) overexpressing ACE2 into the MCT-induced PH rat model, a significant decrease in the expression of pro-inflammatory factors (TNF-α, IL-6, IL-23, IL-17) and a significant increase in the expression of the anti-inflammatory factor IL-10, These findings indicate that hAMSCs overexpressing ACE2 are capable of repairing pulmonary vascular endothelial cell damage by enhancing anti-inflammatory capacity (Wu et al., 2023).

It has been shown that p38 MAPK, c-fos (Briso et al., 2013), iNOS (Zhang et al., 2021) and TNF-α (Zheng et al., 2023b) are all involved in the inflammatory response, and they are widely associated with the pathogenesis of inflammatory diseases, serving a key role in regulating the inflammatory immune process. Combined treatment with lodenafil and hAMSCs reduced the expression of p38 MAPK, c-fos, iNOS, and TNF-α, and reversed functional, structural, and molecular changes through anti-inflammatory and anti-proliferative effects in PH model induced by Su5416/hypoxia (Silva et al., 2020). A variety of research approaches have investigated comparable drug combinations, all validating the therapeutic potential of MSCs in PH (Zapata-Sudo et al., 2020; Zhang et al., 2020b; Liu et al., 2024d). Recent studies have demonstrated that transplanting BMSCs treated with varying concentrations of EPO into MCT-induced PH rat models significantly reduces the expression of TNF-α, IL-1β and IL-6 in lung tissues. Additionally, the relative expression levels of Jagged and Notch1 proteins are significantly reduced. These findings indicate that the combined intervention of EPO and BMSCs confers a protective effect by lowering inflammatory factor levels in lung tissues (Li et al., 2024b).

In addition, the aberrant crosstalk between macrophages and T cells serves as a key driver of immune dysregulation, which constitutes a core pathogenic mechanism underlying PAH. In PAH, there is an increased infiltration of CD68^+^ macrophages accompanied by an imbalance between M1 and M2 subtypes; these macrophages release pro-inflammatory cytokines that exacerbate vascular remodeling. Furthermore, macrophages regulate T cell polarization through the secretion of IL-10, and abnormally activated T cells further amplify pulmonary inflammation (Willis et al., 2018). In MCT-induced PAH models, ADSCs reduce CD68^+^ macrophage infiltration and IL-6 expression, exert anti-inflammatory effects by regulating M1 macrophage phenotypic switching, and alleviate pulmonary vascular remodeling while improving hemodynamics. (De Mendonça et al., 2017). A separate study further demonstrated that HUC-MSCs exhibit superior therapeutic efficacy. Beyond improving right ventricular function and mitigating pulmonary vascular remodeling, HUC-MSCs extensively suppress the infiltration of pulmonary macrophages (both M1 and M2 subtypes) and the expression of related inflammatory cytokines, reduce the recruitment and activation of immune cells such as T cells (Oh et al., 2021).

### 3.5 Increases angiogenic factors

Angiogenesis is governed by a host of “classic” factors—for instance, Vascular endothelial growth factor (VEGF), together with various endogenous “non-classic” peptides such as erythropoietin (EPO) and granulocyte-macrophage colony-stimulating factor (Hoeben et al., 2004). Granulocyte macrophage colony stimulating factor exerts a notable regulatory effect on angiogenesis, and the coordinated regulation of angiogenesis by EPO has likewise been confirmed (Madeddu and Emanueli, 2007; Ribatti and Tamma, 2019). BMSCs pretreated with EPO enhance pulmonary angiogenesis, promote the expression of VEGF, and attenuate vascular remodeling, thereby alleviating right ventricular hypertrophy in rats with PAH (Li et al., 2024b; Zhong et al., 2024). Similarly, Prostaglandin E1 pretreatment increases the protein level of HIF-1α in MSCs, which reduces MSCs apoptosis and enhances the protein levels of CXCR4, MSCs migration, and VEGF secretion. As a result, transplantation of MSCs treated with Prostaglandin E1 alleviates PAH progression by lowering pulmonary artery systolic pressure and the right ventricular hypertrophy index, and by reducing right ventricular failure in the MCT-induced PAH model (Jiang et al., 2022). Schleier Y and colleagues demonstrated that combining granulocyte colony-stimulating factor with BMSCs can reduce the elevation of MCT-induced right ventricular systolic pressure, indicating therapeutic potential for PH treatment (Schleier et al., 2021).

### 3.6 Therapeutic effects of exosomes derived from MSCs on PH

**FIGURE 3:**
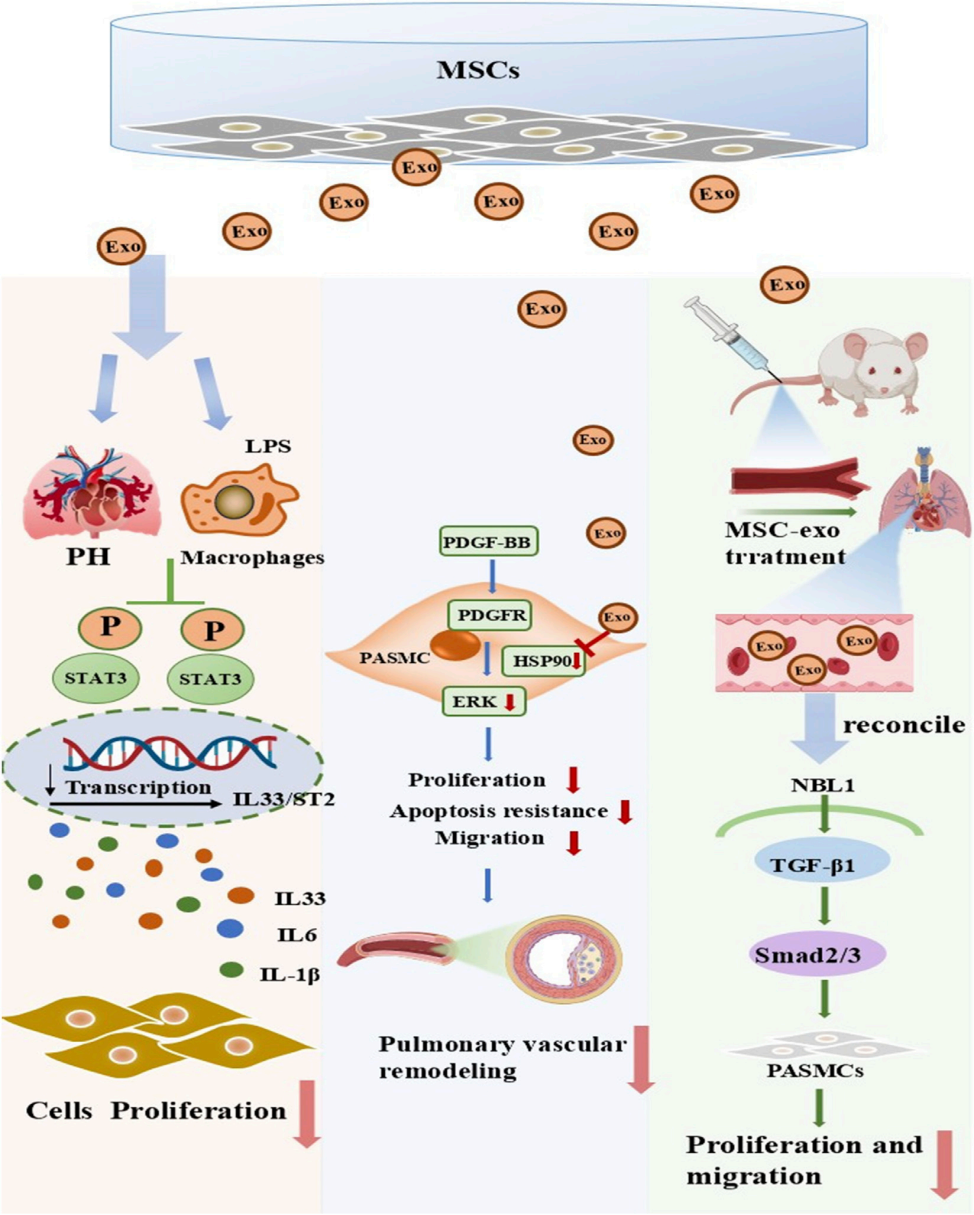
Key mechanisms of inhibition of smooth muscle cell proliferation by exosomes from mesenchymal stem cells. Preclinical studies have proposed that transplanted mesenchymal stem cell-derived exosomes (MSC-Exo) exert beneficial effects in PH through three primary mechanisms. (Ⅰ) MSC-Exo act on macrophages in PH models, inhibiting the activation of the STAT3 pathway and reducing the transcription and release of inflammatory factors such as IL-33, IL-6, and IL-1β, thereby suppressing cell proliferation and alleviating pulmonary tissue inflammatory responses. (Ⅱ) MSC-Exo target the PDGFR receptor on pulmonary artery smooth muscle cells (PASMCs), inhibiting the PDGF-BB/PDGFR/ERK signaling pathway, reducing the proliferation, anti-apoptotic, and migratory capacities of PASMCs, and thus suppressing pulmonary vascular remodeling. (Ⅲ) After being administered to PH model mice, MSC-Exo regulate the NBL1/TGF-β1/Smad2/3 signaling pathway, inhibiting the proliferation and migration of PASMCs and ultimately alleviating pulmonary vascular lesions. PH, pulmonary hypertension; MSCs, mesenchymal stem cells; LPS, lipopolysaccharide; Exo, exosome; PASMCs, pulmonary artery smooth muscle cell.

Exosomes are membranous extracellular vesicles that measure 30–200 nm in diameter (Sokolova et al., 2011). They can be released by most cell types, such as epithelial cells, cancer cells, neural cells, immune cells, embryonic cells, endothelial cells, and MSCs (Mashouri et al., 2019). Compared to other stem cells, MSCs are the most preferred source of therapeutic exosomes (Han et al., 2016). Moreover, compared to viable cell therapy, MSCs-Exo exhibit superior adaptability and greater potential in clinical translation, primarily due to their ultra-small size that enables penetration of the vascular endothelial barrier and interstitial spaces, thereby avoiding the issues of entrapment in pulmonary capillaries (first-pass effect) and mechanical obstruction commonly associated with intravenous infusion of MSCs (Teng et al., 2022; Wei et al., 2022); this allows MSC-Exo to reach target tissues more efficiently while eliminating the potential risks of tumorigenesis and vascular embolism inherent to cell transplantation (Song et al., 2025; Yu et al., 2025). Preclinical studies have demonstrated that MSC-Exo lack major histocompatibility complex -related antigens on their surface, resulting in minimal immunogenicity and enabling cross-species and cross-individual applications, while they can also modulate the functions of immune cells such as macrophages and T cells, inhibit aberrant inflammatory responses, further enhance immunocompatibility, and reduce the risk of rejection (Zhang et al., 2022; Liu et al., 2024c; Yi et al., 2025). Most importantly, MSC-Exo can be isolated in large quantities from multiple MSC sources, including bone marrow, umbilical cord blood, and adipose tissue, and purified via well-established techniques such as ultracentrifugation and tangential flow filtration, facilitating efficient large-scale production (Wang et al., 2020; Liu et al., 2025). They exhibit excellent stability, allowing for cryopreservation and transportation, with well-defined quality control indicators (e.g., particle size, specific markers, and purity) that fully align with the requirements for clinical translation (Rahmatinejad et al., 2024). Notably, they lack the potential risks of long-term poor differentiation and tumorigenesis associated with transplanted cells, rendering their research an active field of investigation. In recent years, a host of studies have indicated that MSC-Exo exhibit favorable therapeutic effects in treating PAH (Ge et al., 2021). Liu’s research has confirmed that MSC injection can effectively reduce macrophage inflammation and alleviate endothelial cell apoptosis in a hypoxia-induced rat model, thereby lowering pulmonary arterial pressure. Additionally, tadalafil pretreatment enhances the secretion of exosomes from MSCs, which in turn increases the expression of miR-29a-3p. This elevation enhances the exosomes’ anti-inflammatory and anti-vascular remodelling properties, and aids in reducing hypoxia-induced smooth muscle cell proliferation and migration (Liu et al., 2024d). In addition, Tail vein injection of HUCMSC-Exo facilitated M2 macrophage polarization, thereby suppressing IL-33/ST2 axis expression and reducing hypoxia-induced pulmonary artery smooth muscle cell proliferation (Liu et al., 2024a). A recent study further demonstrated that BMSC-Exo ameliorated right ventricular hypertrophy and pulmonary vascular remodeling in PH rats by suppressing the Hsp90aa1/ERK/pERK signaling pathway, thereby inhibiting PASMC proliferation (Deng et al., 2024b). Expression attenuate pulmonary vascular remodelling and right ventricular hypertrophy in rats with hypoxic PH by regulating the TGF-β1/Smad2/3 pathway via Nbl1 (Zhang et al., 2024b) (Figure 3). These preclinical studies illustrate the therapeutic potential of MSC-Exo in mitigating PH via their anti-inflammatory and anti-proliferative effects.

In recent years, proteomic and microRNA profiling studies have successfully identified a variety of bioactive molecules in MSC-derived exosomes that are responsible for their therapeutic activity against PH,These molecules include miR-29a-3p (which regulates vascular remodeling) (Liu et al., 2024d), specific miRNAs targeting the EGFR/ErbB2 pathway (Chen et al., 2025), as well as functional proteins such as pyruvate dehydrogenase and glutamate dehydrogenase 1 (which modulate mitochondrial metabolism) (Hogan et al., 2019). By precisely targeting key links in the pathological progression of PH (e.g., vascular smooth muscle cell proliferation, mitochondrial dysfunction, and inflammatory imbalance), these molecules collectively mediate the therapeutic effects of exosomes. Notably, the molecular composition of exosomes exhibits significant “parental cell dependence.” For instance, tadalafil preconditioning can upregulate the expression of miR-29a-3p in MSC-derived exosomes, thereby enhancing their therapeutic efficacy (Liu et al., 2024d). This provides a novel strategy for the targeted enrichment of key bioactive molecules through optimizing parental cell preconditioning protocols. In the future, with the advancement of technologies such as single-cell sequencing and spatial proteomics, it is expected to further decipher the heterogeneity and synergistic mechanisms of bioactive molecules in exosomes, laying a foundation for the development of exosome-based precise targeted therapeutic strategies for PH.

### 3.7 Relief of right ventricular hypertrophy and failure

Vascular and vasoconstriction remodeling lead to a gradual rise in pulmonary vascular resistance (Maron and Leopold, 2015), thus increasing right ventricular afterload. This forces the right ventricle to pump at higher pressures, leading to a rise in mean pulmonary arterial pressure and increased right ventricular systolic wall stress. Ultimately, these changes bring about right ventricular hypertrophy (Hoshijima and Chien, 2002). However, It has been shown that the shift from right ventricular hypertrophy to failure is marked by insufficient angiogenesis (Shiojima, 2005; Izumiya et al., 2006; Bogaard et al., 2009). A clinical trial of MSCs for heart failure in PH is currently underway, and preliminary results indicate that high-dose injections of MSCs are effective in improving cardiac function (Gong et al., 2024). The safety and efficacy assessed in this trial indicate that MSCs play a beneficial role in managing right ventricular failure linked to PH. It is generally accepted that the mechanism by which MSCs alleviate right ventricular (RV) hypertrophy and dysfunction in PH models results from the synergistic effects of paracrine action and reduced pulmonary vascular resistance. Studies have confirmed that administration of adipose-derived mesenchymal stromal cells can promote the secretion of vascular endothelial growth factor (VEGF), attenuate the increase in right ventricular systolic pressure (RVSP) in monocrotaline-induced PH rats, and alleviate RV hypertrophy via paracrine microRNAs (Wang et al., 2019b). Additionally, MSCs locally implanted into the right ventricular wall of PH rats via a novel bioscaffold continuously secrete regenerative/anti-inflammatory factors, inhibiting cardiomyocyte apoptosis, reducing inflammatory infiltration, repairing pulmonary vascular endothelium, suppressing vascular stenosis, and decreasing right ventricular afterload. (Schmuck et al., 2019). Hypoxia-preconditioned BMSCs secrete functional paracrine molecules such as VEGF and miR-126, which not only inhibit right ventricular cardiomyocyte apoptosis, alleviate inflammation and fibrosis, but also improve pulmonary vascular remodeling and reduce mean pulmonary arterial pressure (mPAP) and pulmonary vascular resistance (PVR) (Braga et al., 2023). However, other research findings have demonstrated that ADSCs inhibit the proliferation of pulmonary arterial smooth muscle cells and the deposition of collagen fibers in lung tissues of PAH model rats, alleviate pulmonary vascular remodeling, and mitigate lung tissue inflammation. These effects collectively reduce mPAP and PVR, thereby relieving right ventricular afterload and improving cardiac systolic function (De Mendonça et al., 2017). Collectively, these studies demonstrate that MSCs mitigate RV hypertrophy and dysfunction through two main mechanisms: one involves a “dual targeting” effect mediated by paracrine signaling—directly protecting RV cardiomyocytes and improving myocardial remodeling while indirectly repairing pulmonary blood vessels and reducing RV afterload, with both effects synergistically alleviating RV pathology; the other mechanism focuses on improving pulmonary vascular remodeling and reducing mPAP and pulmonary vascular resistance to relieve RV hypertrophy and dysfunction.

## 4 Factors affecting the efficacy of MSCs therapy for PH

The properties and functions of MSCs are influenced by various factors, including donor age, culture techniques, and preservation methods. The number, proliferation, and differentiation potential of MSCs vary significantly among donors of different ages. For example, research has indicated that the proliferation and differentiation capacities of MSCs derived from older donors are typically weaker than those from younger donors (Kretlow et al., 2008). Additionally, the culture techniques and preservation methods used can also affect the quality and functionality of MSCs, highlighting the importance of standardized protocols in research and clinical applications (Stolzing et al., 2008). The senescence of MSCs during *in vitro* culture and expansion is a key factor impacting their therapeutic effectiveness. As MSCs undergo multiple rounds of expansion, their capacity to differentiate into adipogenic and osteogenic lineages tends to diminish (Yang et al., 2018). Over the last decade, the culture approach for MSCs has advanced from conventional two-dimensional (2D) to three-dimensional (3D) culture systems. This transition has qualitatively improved the differentiation, proliferation, and secretion of paracrine factors by MSCs. 3D culture provides a more physiological microenvironment, leading to enhance production of signaling molecules and improve therapeutic potential (Qi et al., 2024; Zhu et al., 2024b). Additionally, cell sheets were prepared using poly N-isopropylacrylamide modified cell culture dishes. This method employs a gradient cooling technique to collect the cell sheets, thereby avoiding the damage to cell activity that can result from trypsin digestion. Consequently, the structural integrity and function of the cells are preserved (Nakao et al., 2019). Recent studies show that MSCs sheets display strengthened cell adhesion and upregulate key cytokines (such as IL-10, TGF-β1, HGF, and IL-6), and show superior functional performance compared to conventional culture methods (Nakao and Nagase, 2024). Therefore, choosing a suitable cell collection method is vital for the success of stem cell therapy. Preservation methods for MSCs have also been a significant challenge that researchers have addressed in recent years. The conventional cryoprotective agent is dimethyl sulfoxide; however, recent clinical studies have started using cryoprotective agents free of dimethyl sulfoxide to maintain the genetic integrity of MSCs. This is because some research has indicated that dimethyl sulfoxide might trigger genetic changes in MSCs (Wang and Li, 2024), Zhang et al. demonstrated that cryopreservation with 1.0 M trehalose (Tre) and 20% glycerol significantly improved post-thaw viability and proliferative capacity of human adipose-derived MSCs compared to conventional cryopreservation approaches (Zhang et al., 2020c). Recently, it was reported that cell viability, adhesion, and trilineage differentiation potential can be preserved using a cryoprotection solution composed of 90% fetal bovine serum and 10% dimethyl sulfoxide (Abraham and Goel, 2024). Ongoing refinement of extraction and culture techniques has allowed MSCs to yield remarkable outcomes in treating a broad spectrum of diseases. This advancement paves the way for MSCs to be applied in addressing numerous refractory conditions.

## 5 Issues to be addressed in the treatment of PH by MSCs

MSCs have started to demonstrate therapeutic potential in clinical trials for diverse diseases, such as neurodegenerative ones (Rash et al., 2025), cardiovascular, and autoimmune diseases (Shan et al., 2024). These findings also reveal the clinical safety of MSCs. Consequently, clinical studies of MSCs for PH treatment have gradually become a focus in recent years. Moreover, MSCs are more clinically acceptable due to their high histocompatibility and low immunogenicity, and most importantly, they are easy and safe to obtain without major ethical issues (Wang et al., 2014). These strengths render MSCs a promising tool for clinical use. Nevertheless, despite the encouraging outcomes of MSCs in diverse preclinical disease models, clinical trials employing MSCs across various medical conditions have fallen short of expectations. This is attributed in part to the following disadvantages of MSCs; (1) intrinsic heterogeneity (Lyu et al., 2024), (2) Potential risk of developing tumors (Xie et al., 2020), (3) Decreased cellular phenotype and biological function (Wong et al., 2021) (4) Low post-transplant survival (Guo et al., 2020). These drawbacks impede the further clinical application of MSCs. MSCs themselves form a heterogeneous population, with their variability primarily manifested in proliferative capacity, differentiation potential, and immunomodulatory capability (Elahi et al., 2016). The heterogeneity of MSCs is affected by multiple factors, such as the tissue origin and donor age, which can diminish the therapeutic effectiveness of MSCs transplanted in clinical environments (Heng et al., 2014). In recent years, the rapid advancement of single-cell RNA sequencing (scRNA-seq) technology and specific surface marker analysis has provided an innovative solution to address the key bottleneck of MSC heterogeneity, which restricts the stability of therapeutic efficacy in the treatment of PAH. Through scRNA-seq technology, researchers have successfully deciphered the subpopulation composition and functional specificity of BMSCs and HUC-MSCs, and identified a dominant subpopulation with therapeutic potential for PAH (Xie et al., 2022; Cyr-Depauw et al., 2024). This includes Fibro-MSCs with high proliferative capacity and multilineage differentiation potential in BMSCs (Bandyopadhyay et al., 2024), as well as two key subpopulations (progenitor cell-like and fibroblast-like) in HUC-MSCs (Cyr-Depauw et al., 2024). In terms of surface marker screening, a recent study identified that the Podoplanin^+^CD36^+^ subpopulation highly expresses proliferation- and migration-related markers, thereby providing a basis for the precise screening and culture optimization of placental mesenchymal stromal cell subpopulations (Boss et al., 2022),and the combined application of CD73/CD105 and functional genetic markers (BAMBI, MFGE8) enables the precise enrichment of homogeneous MSC subpopulations relevant to PAH therapy (Chen et al., 2022). These research advances not only clarify the functional specificity and sorting feasibility of MSC subpopulations but also provide crucial theoretical basis and technical support for the transformation of MSC-based PAH therapy toward precision and standardization, holding significant implications for enhancing the stability of clinical therapeutic outcomes. Although pretreatment with inflammatory factors such as IFNγ and TNFα has been shown to lead to consistent gene expression in MSCs (López-García and Castro-Manrreza, 2021), more methods to reduce heterogeneity still need to be continuously explored and developed. Furthermore, the problem of tumorigenicity, which has long plagued researchers, remains a major obstacle in the clinical use of MSCs. To reduce the potential risk of tumor formation, the number of MSCs used in therapy should be minimized. Beyond reducing cell numbers, Qi et al. investigated the positive effect of culturing MSCs under 3D and hypoxic conditions (3D_Hypo MSCs), which revealed the potential applicability of this method for clinical cell therapy applications (Qi et al., 2024). Traits associated with MSCs, such as proliferation rate, differentiation potential, and immunomodulatory functions, often decrease significantly during cell extraction and culture (Liu et al., 2020a). Nevertheless, a number of potential approaches to stabilize the biological functions of MSCs during *in vitro* expansion have been documented in recent years. Instances include gene editing and specific combinations of small molecule compounds (Gruenloh et al., 2011), and research has explored the use of embryonic stem cells or induced pluripotent stem cells—expanded *in vitro* and then induced to differentiate into homogeneous MSCs (Lotfinia et al., 2016; Luzzani and Miriuka, 2017). Furthermore, the stabilization of MSCs during *in vitro* amplification has been accomplished by boosting their biological functions through a GMP-grade three-dimensional hypoxic mass production system (Qi et al., 2024). Whether the viability and functional integrity of MSCs can be maintained *in vivo* post-transplantation is also one of the key challenges that hinder the full exertion of their therapeutic efficacy. In recent years, researchers have conducted extensive explorations to address the challenge of MSC survival after transplantation. For instance, hypoxic preconditioning under 1% O_2_ can significantly enhance the paracrine function of MSCs (e.g., upregulating the secretion level of VEGF), thereby improving their therapeutic efficacy in the pathological microenvironment (Ho et al., 2018; Ishiuchi et al., 2020). Multifactorial preconditioning of MSCs with interferon-γ (IFN-γ), TNF-α, and IL-1β can induce the generation of regulatory T cells, enabling MSCs to rapidly respond to inflammatory signals, strengthen anti-inflammatory effects, and further enhance their immunomodulatory function (Strauch et al., 2020; Hackel et al., 2023). The application of 3D culture technology has emerged as a key breakthrough. This technology not only reduces the extracorporeal loss of MSCs after infusion but also optimizes their paracrine function, while decreasing the apoptotic risk of MSCs themselves in the pathological environment, thereby effectively maintaining cell quantity and functional integrity (Kim et al., 2022; Dai et al., 2024; Song et al., 2025). In addition, researchers have further identified that multiple approaches, such as granulocyte (Frid et al., 2024), protection of macrophages from pyroptosis (Zhang et al., 2024a), promotion of glutathione synthesis (Yu et al., 2024), and α-ketoglutaric acid pretreatment (Li et al., 2024c), can effectively enhance the survival rate of stem cells following transplantation. But these approaches have limitations in practical clinical use. Hence, identifying simpler and more efficient means to boost the survival rate of cell transplantation is vital for improving therapeutic efficacy. In summary, reducing the heterogeneity and tumorigenicity of MSCs, stabilizing their biological functions, and improving post-transplantation survival rates are key challenges that researchers must address to maximize their therapeutic potential in clinical applications.

Currently, preclinical studies on PH models have initially verified the long-term safety of MSC therapy, with no evidence indicating that MSC administration induces ectopic tissue formation, chromosomal instability, fibrosis nodules, or other adverse events. However, it is important to objectively note that existing research still has limitations, including the lack of large-sample, long-duration dedicated safety studies. Meanwhile, MSC therapy research in other diseases has highlighted relevant potential risks (Tarte et al., 2010; Berardis et al., 2015). Therefore, in the context of MSC therapy for PH, efforts should be directed towards two key aspects: on the one hand, reducing MSC heterogeneity and tumorigenicity, stabilizing the biological functions of MSCs, and improving cell survival rate after transplantation; on the other hand, standardizing cell culture processes and administration protocols to mitigate risks from an operational perspective, conducting targeted long-term safety assessments, and further improving the risk evaluation system of this therapy. These measures will provide a more solid safety basis for clinical translation.

## 6 Conclusion and perspectives

Over the past few decades, thanks to the efforts of countless researchers, substantial advancements have been made in unraveling the mechanisms underlying PH progression. Although PH remains incurable, these studies have facilitated more effective management of the condition. This review underscores the significant potential of MSCs to ease right ventricular hypertrophy, suppress smooth muscle cell proliferation and pulmonary artery vascular remodeling, ameliorate pulmonary vascular endothelial dysfunction, and diminish anti-inflammatory factor expression. Despite the promising therapeutic potential of MSCs for PH, broader large-scale production and clinical application require addressing key challenges of MSCs—including heterogeneity, tumorigenicity, reduced biological function, and senescence during *in vitro* culture—through the following strategies: standardizing MSC preparation and enriching homogeneous subpopulations using flow cytometry and immunomagnetic bead sorting; mitigating safety risks via small-molecule intervention, gene editing, and enhanced tumorigenicity testing; optimizing the 3D dynamic culture system, supplementing with growth factors and antioxidants, and combining gene modification or pharmaceutical intervention to delay senescence and functional decline. In addition, three critical issues still require further refinement. First, there is a lack of unified standards for the isolation and purification techniques of MSC-derived exosomes, as well as quality control indicators such as particle size and specific markers. Future multi-center collaborative studies are needed to identify the optimal preparation processes for exosomes from different sources. Second, the targeting ability and functional efficiency of MSCs need to be enhanced. Future studies should focus on the key pathological pathways of PH, screen specific microRNAs for MSC modification, and improve the pulmonary vascular-targeted homing capacity of MSCs via genetic engineering technologies. Third, after intravenous infusion, MSCs are prone to entrapment in pulmonary capillaries or clearance by the immune system, resulting in a low colonization rate in target organs. Future efforts should focus on developing biomaterial-based delivery vectors, combined with local delivery approaches like intratracheal instillation. It is worth noting that MSCs therapy offers hope to patients who do not respond well to traditional drugs, thus meriting further exploration of its therapeutic potential. In conclusion, further research is needed to fully elucidate the mechanisms, safety, efficacy, and optimization of MSC therapy in PH for clinical application.

## Abbreviations

ADSCs, Adipose mesenchymal stem cells; ACE2, Angiotensin-converting enzyme 2; BMSCs, Bone marrow mesenchymal stem cells; CM, Conditioned medium; EMT, Endothelial mesenchymal transition; EVs, Ectodermal vesicle; eNOS, Endothelial nitric oxide synthase; Exo, Exosome; EPO, Erythropoietin; hAMSCs, Human amniotic mesenchymal stem cells; HUC-MSCs, Human umbilical cord mesenchymal stem cells; ISCT, International Society for Cellular Therapy; MSCs, Mesenchymal stem cells; MV, Microvesicle; MCT, Monocrotaline; NF-κB, Nuclear factor kappa-B; NVs, Nano-vesicle; PH, Pulmonary hypertension; PAH, Pulmonary arterial hypertension; PASMCs, Pulmonary artery smooth muscle cells; SuHx, SU5416 combined with hypoxia; VEGF, Vascular endothelial growth factor.
